# Faulty Environment

**Published:** 1902-01

**Authors:** E. K. Wedelstaedt

**Affiliations:** St. Paul, Minn.


### International Dental Journal.

Vol. XXIII. January, 1902. No. 1.

### Original Communications.[1]

### FAULTY ENVIRONMENT.[2]

BY E. K. WEDELSTAEDT, ST. PAUL, MINN.

Mr. President and Gentlemen,—As the choice of a topic was left to me, I felt that some good might result if I tried to interest you in faulty environment,—*i.e.*, the leaving of opportunities that breed disease in teeth we fill or in surrounding parts.

It is out of the question to go over this whole field in a single essay. I have written two other essays on this topic, but in none are the same cases cited. The conditions, however, are similar in all the cases.

To fix this subject firmly in our minds, let me speak of a case from practice. Mrs. A. came to me for the purpose of having her teeth examined. She informed me that for ten years she had been immune to caries. Her age was about forty-five. There were thirty-two teeth in her mouth, thirteen of which contained nineteen fillings. All the fillings had been made with gold, save a cavity in the occlusal surface of a lower right third molar, which

[1] The editor and publishers are not responsible for the views of authors of papers published in this department, nor for any claim to novelty, or otherwise, that may be made by them. No papers will be received for this department that have appeared in any other journal published in the country.

[2] Read before the American Academy of Dental Science, Boston, October 2, 1901.

contained an amalgam filling. There was a considerable amount of calculus on the lingual surfaces of the ten lower teeth: more calculus on the upper teeth on the left side than on the upper teeth on the right. Looking for the cause, I found a faulty inter-proximal space between the upper left bicuspids, and another between the second bicuspid and molar. The mesial surface of the upper left first molar had been Arthurized. The mesial surface contained a gold filling, 3.2 millimeters in diameter, and it had been made in a round hole. The gum in the vicinity of the bicuspids and molar was of a purplish color, and the amount of tissue involved can be better imagined if I say that it covered a space equal to a dime cut in halves. I called the woman's attention to the spaces and the color of the gum, and compared these with the conditions existing on the right side. I told her I was quite certain that at present little use could be made of the teeth on the left side; but if an alteration were made in the conditions, I felt it would lead to much more comfort. She answered that she was entirely satisfied with the conditions as they were, and that about all her mastication was done on the left side of her mouth. She had no trouble with the food crowding into the spaces. After some talk, however, she reluctantly consented to have the necessary work made. The second bicuspid was moved into its normal position and then the mesial surface of the molar filled, thus restoring it to its original mesio-distal diameter. This work took some time; and when all was completed, I requested that a report be given me in about two weeks. In three weeks or so the woman returned and said, "When I told you that I did all my chewing on the left side and that the food did not annoy me, I misinformed you. For a number of years I have not made any use of the teeth on that side. The food would crowd into these spaces, and it annoyed me to such an extent that I was driven nearly frantic. But since this tooth has been filled I have had comfort, and it is a pleasure for me to eat on that side." Now here was a typical case of faulty environment, and one, except the misrepresentation by the patient, of a very common variety. For a few moments let me try to interest you in this particular case, and then I can touch on other conditions.

In Arthurizing the mesial surface of the upper left first molar, the cutting had been sufficient to remove the enamel from the entire mesial surface, or from the gingival margin to the occlusal surface. It was 7.25 millimetres from the lingual to the buccal enamel margin. Thus there was that extent of dentine exposed to the action of the micro-organisms. There was a shoulder left at the gingival margin, for contact, I suppose. When the tooth was ready for work, the rubber dam was adjusted, the gold filling dislodged, and then the disintegrated dentine removed until sound tissue was reached. This sound tissue had for its lingual and buccal cavity margins the enamel as originally left, and the gingival cavity margin was in the cementum. The anchorage was placed in the central pit, making an occlusal anchorage which was at right angles with the cavity proper.

We know definitely several things:

*First.* That wherever teeth are Arthurized and the enamel removed from a proximal surface, it is but a question of time before the dentine disintegrates.

*Secondly.* That wherever the teeth in the arch are normally situated, any operation we may make in their proximal surfaces which does not return the parts to their normal condition results in faulty environment; therefore, faulty environment I should define as any abnormal condition which brings contiguous parts into improper relations.

*Thirdly.* With the development of our knowledge, we become certain that faulty environment breeds disease. With our knowledge of conditions developed to this extent, will somebody have the kindness to tell me why it is that some men in our profession still call attention to the beautiful results that follow the application of the Arthur method? Within fifteen months I heard a man advocate the use of this method. He alleged that he had patients whose teeth had remained in first-class condition for thirty or forty years after being Arthurized. I cannot believe that the teeth of those who live in the East are any different from the teeth of those who live in the West; but I do believe that the teeth of all individuals under the same conditions and environment suffer about the same.

In all operations which are made in the proximal surfaces of teeth,—I am speaking now of teeth that are normally situated,—it is essential that we restore the teeth to their original mesio-distal diameter, so that the interproximal space can be restored to its original size. Space in which to work must be obtained so that we can restore the parts to their original size. How this space is obtained makes little difference, so that the means used do not inflict needless injury to the gum septum. (I trust that it is not necessary for me to refer to the barbarous method so long in vogue, of driving wooden wedges for the purpose of obtaining immediate separation. That method has long since been given up by the more intelligent class in our profession.) Obtain space so that all operations on the proximal surfaces of teeth can be made properly. If you cannot obtain space, then do not do the work. Remember that surgeons will not perform deliberate operations when constitutional conditions are unfit. Neither should we make any operations unless the conditions are such that lasting benefit will result to the patient. I am well aware that space in which to work is not obtained by many operators; but that is no excuse for our not obtaining it. We would wish our own teeth filled properly, so let us fill the teeth of our patients just the same as we would wish our own filled. I see a great many fillings which have been made by men in different sections of this land, and I am often amazed at the conditions that confront me. When I say to you that more failures can be charged directly to the leaving of faulty environment in filling teeth than to any other one cause, I feel certain I merely echo the sentiments of many intelligent men who are doing all possible to interest dentists in doing away with these faulty conditions. Let me speak of some of these conditions. I find an almost general disregard of the necessity for

(1) The application of the extension for prevention method;
(2) A proper contour for the interproximal space;
(3) Trimming properly all metal fillings, and
(4) Placing contact points on fillings or crowns.

I could name a number of other conditions, but these are more than enough for us to consider. Why, let me ask you, is there such failure in giving attention to these different things? But this is not the time or place for me to do otherwise than consider very briefly the four conditions which have just been named.

1. The necessity for the application of the extension for prevention methods. The *Items of Interest* for May, 1901, and the *Review* for August, 1901, contain my views regarding this subject. I have no desire to alter them; and, further, I do not wish to take up your time in arguing for a teaching which, I feel, should be followed by all dentists who have at heart the welfare of those whom they serve, and who are willing to study the conditions which lead to failure. I shall close my remarks on this topic by asking those who do not believe in this teaching to make an experiment, which will quickly set at rest any doubts which they may have regarding the necessity for applying this method. Let an unbeliever, when working for a patient he can keep track of, and in whose mouth new cavities of decay are occurring, take four cavities that are in the proximal surfaces of bicuspids or molars. Let him apply the extension for prevention method to two cavities, and not apply it to the other cavities. Within five years the results that will follow will make him a believer in this theory, for at the end of this time recurrence of decay will generally be found in the two cases last mentioned, due wholly to faulty environment. (I am not speaking of isolated teeth, but of those which have adjoining teeth in position.) When an operator has made a number of such experiments, and has become familiar with the results that follow, he is in a position to speak authoritatively regarding the necessity of following certain well-known and definite methods to which many are now calling attention. I am most desirous of emphasizing the importance of this subject, to the end that more men will take an interest in it, and obtain more definite knowledge regarding the necessity for studying the conditions which lead to the failure of so many fillings in the proximal surfaces of teeth. There is no need of so many of these failures. Many people lose confidence in our ability. There should be a general effort made to eradicate this evil and to further improve the fair name and fame of American dentists.

2. The necessity of a proper contour for the interproximal space. I do not know how to consider this topic very briefly, but let me cite two cases for your consideration. Both operations were made by well-known professors of operative dentistry in our colleges. One case was a gold filling in the mesio-incisal surface of an upper left central incisor. No fault could be found with the cavity preparation. When the filling was finished, I was requested to inspect it; I was not asked to criticise. I found a beautifully finished filling, imperfect, however, in contour. The mesio-distal diameter of the tooth had been reduced about one-fifth. If a small broach had been placed mesio-distally in the space, about one-third the length of the tooth towards the gingival from the incisal surface, we would have had an almost perfect capital letter A. Now this operation was made for a woman who, every time she spoke, showed, let me in all charity say, the work of an ignorant man. Later, I shall consider this case further.

The other operation was a very large gold filling in the disto-occlusal surface of an upper right first molar. What a beautiful operation it was, in so far as finish and cavity preparation were concerned! The lingual and the buccal enamel margins were extended just far enough, but the gingival margin of the filling was covered with an unhealthy gum septum. Every part of the operation was carried through with a degree of faithfulness that is seldom seen. But the man had no space in which to work. The tooth was not restored to its original size, nor was the interproximal space restored. And the contact point,—well, he had placed it in the middle third of the filling.

Let us take these two cases and examine the conditions. Here we have the two extremes of faulty environment. One case is as bad as the other, and leads to equally injurious results. Food crowds into these spaces; it is held there; it undergoes decomposition, and it keeps the mouth in an unhygienic condition. This leads to functional disarrangement through the loss of the use of the teeth. It is our duty to correct these evil conditions, returning the parts to as nearly a normal condition as lies in our power. It is not often accomplished without hard and unremitting toil. Still, we should meet these conditions fairly and aim to do away with them entirely. I should like to say more to you about this subject, but you are intelligent men, and the plans for doing this work have been well presented for some time past.

3. The necessity for trimming properly all metal fillings.

Of all the work which we are called upon to do, I know of none that so tries my patience as that of properly trimming the proximal surface of fillings. It does not make any difference how perfect all the work has been made up to this point; it is useless if that proximal surface is not perfectly trimmed. If we leave overhanging margins, yeast-ditches (Searl), little grooves and hollows in the filling or around it, then we are leaving opportunities for fermentation to undo our work. There is no other part of the operation that is so little understood by dentists generally. If I am to judge from the condition of the fillings which come to me from other practitioners, I am compelled to say that there are too many men who seem to believe that all that is necessary to do is to *fill* the teeth of their patients. It does seem that a little more attention should be given this subject. I am opposed to this habit of leaving so many fillings unfinished. A most serious condition of affairs confronts us, and it is one, too, that results in much more harm than many seem to suppose.

Regarding this matter of trimming fillings, let me say that there are other things besides disks and strips for trimming the proximal surfaces of fillings. I should like to call your attention to some of the other things. Here is a Black saw, some Black files and knives. These instruments are used for trimming the filling to form on the proximal surface. The saw is used for removing the surplus gold which lies along the gingival margin. I use it in this way: after the completion of the marginal condensation I loosen the saw from the frame and shove one end through the interproximal space. It is then replaced in the frame, and this nut on the end turned until the saw is rigid. The saw is then gently insinuated under the overhanging filling-material, and, cutting towards the occlusal, the surplus is removed. I do not use the saw more than once. I make an attempt to remove about the gingival fifth of the surplus. I do not continue the use of the saw until there is an opening into the interproximal space from the occlusal surface. These files follow the use of the saw, and the knives are used after the files. The filling-material on the proximal surface is usually completely trimmed with these instruments. When the trimming is completed, strips and disks are used to remove the file-marks and to prepare the filling for the final polish. It is permissible to use rotary disks, provided that they are used to the lingual and to the buccal of the contact point. But if there is an adjoining tooth in position, they should never be passed into the interproximal space from the occlusal surface. If this is done, the contact point on the filling is removed, and another must be made if the filling is to leave our hands in the best condition possible. I have absolutely no patience with those writers on dental topics who assert that they can and do trim and finish all fillings made in the proximal surfaces of teeth by using the rotary disks alone. I have seen many fillings that have been made by some men who have made this assertion, and the less said about their fillings the better for us all. If we will but bear in mind that there are instruments for us to use when fillings are to be trimmed, and polishing strips for polishing fillings, and use them for the purpose for which they are intended, we shall very quickly observe how much more rapidly we can do our work. Nor is this all. The improvement in shaping our fillings will be so great that we may wonder why it was that we did not make use of these instruments at an earlier period.

4. The necessity for placing contact points on fillings and crowns.

I have spoken of the necessity for obtaining space where operations were to be made in the proximal surfaces of teeth, and I have dwelt on its importance. Without space in which to finish fillings, the interproximal space is not restored to its normal condition, nor is the mesio-distal diameter of the tooth returned to its normal size. We must have space to properly contour and finish all fillings made in proximal surfaces. If we have not space in which to do these things, how is it possible for us to make a contact point on a filling or crown? And if a tooth cannot be properly restored, then, I say, do not do the work.

Large contact points and those placed in the middle or gingival third of the proximal surface lead to irritation and the absorption of the gum in the interproximal space, and all three of these conditions are simply opportunities which breed disease. Those who have somewhat different ideas regarding this subject are requested to study with care the faceted contact points worn on the mesial and distal surfaces of many teeth. Obtain a selection of extracted teeth and examine the condition of the proximal surfaces. You may be surprised at some of the things which will be revealed to you. The teeth and their arrangement in the arch must be studied if we wish to fully understand what constitutes a normal proximal surface. Thereafter, if in making repairs we return the proximal surfaces to as near a normal condition as possible, we shall have done our whole duty, provided that all the other work has been made as thoroughly. It is impossible for me to emphasize too much the necessity for studying carefully the conditions with which we deal, so that we may be in a position to return any part to its normal condition when corrective measures are necessary.

I have touched upon these different subjects very briefly, and I can only ask that some effort be made by all to obtain some comprehensive knowledge of these different things by a comparison of the normal with the abnormal conditions. This comparison will very quickly prove how necessary it is to make all contact points as small as possible, and to have them placed properly on all fillings and crowns.

As it is, this essay is too long, and I must close without saying one-quarter of what I feel is necessary about the different things I have asked you to consider.

I have simply emphasized the importance of our considering with a little more care a few teachings, and have given you the benefit of my ideas. I should like very much to have the benefit of your ideas, for with an exchange of thoughts much is learned; otherwise, nothing is gained.

